# Re: Mini incision open pyeloplasty – Improvement in patient outcome

**DOI:** 10.1590/S1677-5538.IBJU.2015.0616

**Published:** 2015

**Authors:** Vishwajeet Singh, Manish Garg, Pradeep Sharma, Rahul Janak Sinha, Manoj Kumar

**Affiliations:** 1 Department of Urology, King George Medical University, Chhatrapati Shahuji Maharaj Medical, University), Lucknow, India

*To the editor,*

The authors of “Mini incision open pyeloplasty - Improvement in patient outcome” are to be congratulated for publishing their work in the era of minimally invasive surgery (MIS) (1). Refinement of open surgery should still be ongoing and published because not every surgeon can do MIS, and not every patient can afford MIS which is more expensive than that of open surgery.

However, two issues need to be clarified. The first issue is how to select the right patient. Most of the patients presented as lumbar pain and hydronephrosis. Is the pain related to hydronephrosis/UPJO? The mean T1/2 of diuretic renal scan was 26.7±6.4 minutes. It means that some of the patients actually had a T1/2 shorter than 20 minutes. What were the surgical indications for this group of patients?

The second issue is how to define success? The authors reported an overall success rate of 98.6% without clear definition. By grade of ultrasound, there were at 5 patients (7.0%) with postoperative grade 3–4 hydronephrosis; by grade of IVP, there were 7 patients (10%) with moderate to severe hydronephrosis (table 2). Could we define these as success? T1/2 was greatly improved postoperatively. However, how many of them still had obstructive curve or T1/2 >20 minutes?

*CHen Ke-Chi, MD* *Yang Stephen Shei-Dei, MD* *Division of Urology* *Buddhist Tzu Chi General Hospital* *Taipei Branch, Taipei Taiwan* *Medical College of Buddhist Tzu Chi University* *Hualien, Taiwan* *Fax: + 88 623 366-8042* *E-mail: krissygnet@yahoo.com.tw*
